# BRET-Based Self-Cleaving Biosensors for SARS-CoV-2 3CLpro Inhibitor Discovery

**DOI:** 10.1128/spectrum.02559-21

**Published:** 2022-06-27

**Authors:** Ningke Hou, Chen Peng, Lijing Zhang, Yuyao Zhu, Qi Hu

**Affiliations:** a Key Laboratory of Structural Biology of Zhejiang Province, School of Life Sciences, Westlake University; Center for Infectious Disease Research, Westlake Laboratory of Life Sciences and Biomedicine; and Institute of Biology, Westlake Institute for Advanced Study, Hangzhou, Zhejiang, China; CIRI; Université de Lyon; Inserm U1111

**Keywords:** 3C-like protease, BRET, SARS-CoV-2, inhibitor, self-cleaving biosensor

## Abstract

The 3C-like protease (3CLpro) of SARS-CoV-2 is an attractive drug target for developing antivirals against SARS-CoV-2. A few small molecule inhibitors of 3CLpro are in clinical trials for COVID-19 treatments, and more inhibitors are under development. One limiting factor for 3CLpro inhibitors development is that the cellular activities of such inhibitors should be evaluated in Biosafety Level 3 (BSL-3) laboratories. Here, we design DNA-coded biosensors that can be used in BSL-2 laboratories to set up cell-based assays for 3CLpro inhibitor discovery. The biosensors were constructed by linking a green fluorescent protein (GFP2) to the N-terminus and a Renilla luciferase (RLuc8) to the C-terminus of SARS-CoV-2 3CLpro, with the linkers derived from the cleavage sequences of 3CLpro. After overexpression of the biosensors in human embryonic kidney (HEK) 293T cells, 3CLpro can be released from GFP2 and RLuc by self-cleavage, resulting in a decrease of the bioluminescence resonance energy transfer (BRET) signal. Using one of these biosensors, pBRET-10, we evaluated the cellular activities of several 3CLpro inhibitors. These inhibitors restored the BRET signal by blocking the proteolysis of pBRET-10, and their relative activities measured using pBRET-10 were consistent with their previously reported anti-SARS-CoV-2 activities. We conclude that the biosensor pBRET-10 is a useful tool for SARS-CoV-2 3CLpro inhibitor discovery.

**IMPORTANCE** The virus proteases 3CLpro are validated drug targets for developing antivirals to treat coronavirus diseases, such as COVID-19. However, the development of 3CLpro inhibitors relies heavily on BSL-3 laboratories. Here, we report a series of BRET-based self-cleaving biosensors that can be used to set up cell-based assays to evaluate the cell permeability and cellular activity of SARS-CoV-2 3CLpro inhibitors in BSL-2 laboratories. The cell-based assay is suitable for high-throughput screening for 3CLpro inhibitors because of the simplicity and good reproducibility of our biosensors. The design strategy can also be used to design biosensors for other viral proteases for which the activation processes involve the self-cleavage of polyproteins.

## INTRODUCTION

The severe acute respiratory syndrome coronavirus 2 (SARS-CoV-2), which caused the global pandemic of COVID-19, poses a great threat to public health (1). Despite several vaccines being accessible, effective antivirals are still urgently needed for the treatment of COVID-19 (2). The RNA genome of SARS-CoV-2 encodes two large overlapping polyproteins, pp1a and pp1ab, as well as several structural proteins and accessory proteins (3). During virus replication in host cells, pp1a and pp1ab are expressed and then cleaved to generate 16 nonstructural proteins (nsps). The cleavages are catalyzed by nsp3 and nsp5, two proteases included in the 16 nsps. Specifically, the papain-like protease (PLpro) domain of nsp3 cleaves the peptides bonds between nsp1 and nsp2, nsp2 and nsp3, and nsp3 and nsp4, while the peptide bonds between other nsps are cleaved by nsp5 (also called 3C-like protease, 3CLpro, or the main protease). Inhibition of 3CLpro is an effective strategy to develop antivirals against SARS-CoV-2 (4). Several 3CLpro inhibitors have been reported, two of which are now undergoing COVID-19 clinical trials (5–11).

Enzymatic assays using purified 3CLpro were frequently used in the initial screening of 3CLpro inhibitors, but to evaluate the cell permeability and cellular activities of the inhibitors, cell-based antiviral assays are necessary. The requirement of Biosafety Level 3 (BSL-3) for performing the cell-based anti-SARS-CoV-2 assays has slowed the development of 3CLpro inhibitors.

To set up cell-based 3CLpro assays that can be done in BSL-2 laboratories, four types of biosensors have been developed. The first is luciferase-based biosensors that have a 3CLpro cleavage site inserted into a circularly permuted luciferase. Upon cleavage by 3CLpro, the luciferase is activated (12, 13). A similar idea has been used to develop a GFP-based 3CLpro biosensor (14, 15). The second is also a luciferase-based biosensor with two complementary luciferase fragments linked by a 3CLpro cleavage site, but the luminescence is lost by 3CLpro cleavage and restored when 3CLpro activity is inhibited (16). The third is a GFP fusion protein having an ER targeting domain linked to the C-terminus of the GFP through a 3CLpro cleavage site; 3CLpro-catalyzed cleavage leads to a translocation of the GFP from the ER to the nucleus, which can be quantified using light microscopy (17). The fourth is a biosensor in which a Src myristoylation domain and an HIV-1 Tat-GFP fusion protein are linked to the N- and C-terminus of 3CLpro, respectively, through 3CLpro cleavage sites. Expression of this biosensor in HEK 293T cells showed little GFP fluorescence, while inhibition of 3CLpro greatly increased the GFP fluorescence, probably because 3CLpro-catalyzed self-cleavage led to the degradation of the biosensor (18).

A limitation of these biosensors are that their readouts are highly dependent on the expression levels of the biosensors, and for the first three types of biosensors, the expression level of 3CLpro also affects the readouts. Another limitation is that the first three types of biosensors require either cotransfection of two plasmids (the biosensor and 3CLpro plasmids) or transfection of 3CLpro into cells stably expressing the biosensors. The fourth type only needs to transfect one plasmid, but its sensitivity to 3CLpro inhibitor GC376 is much lower.

**FIG 1:**
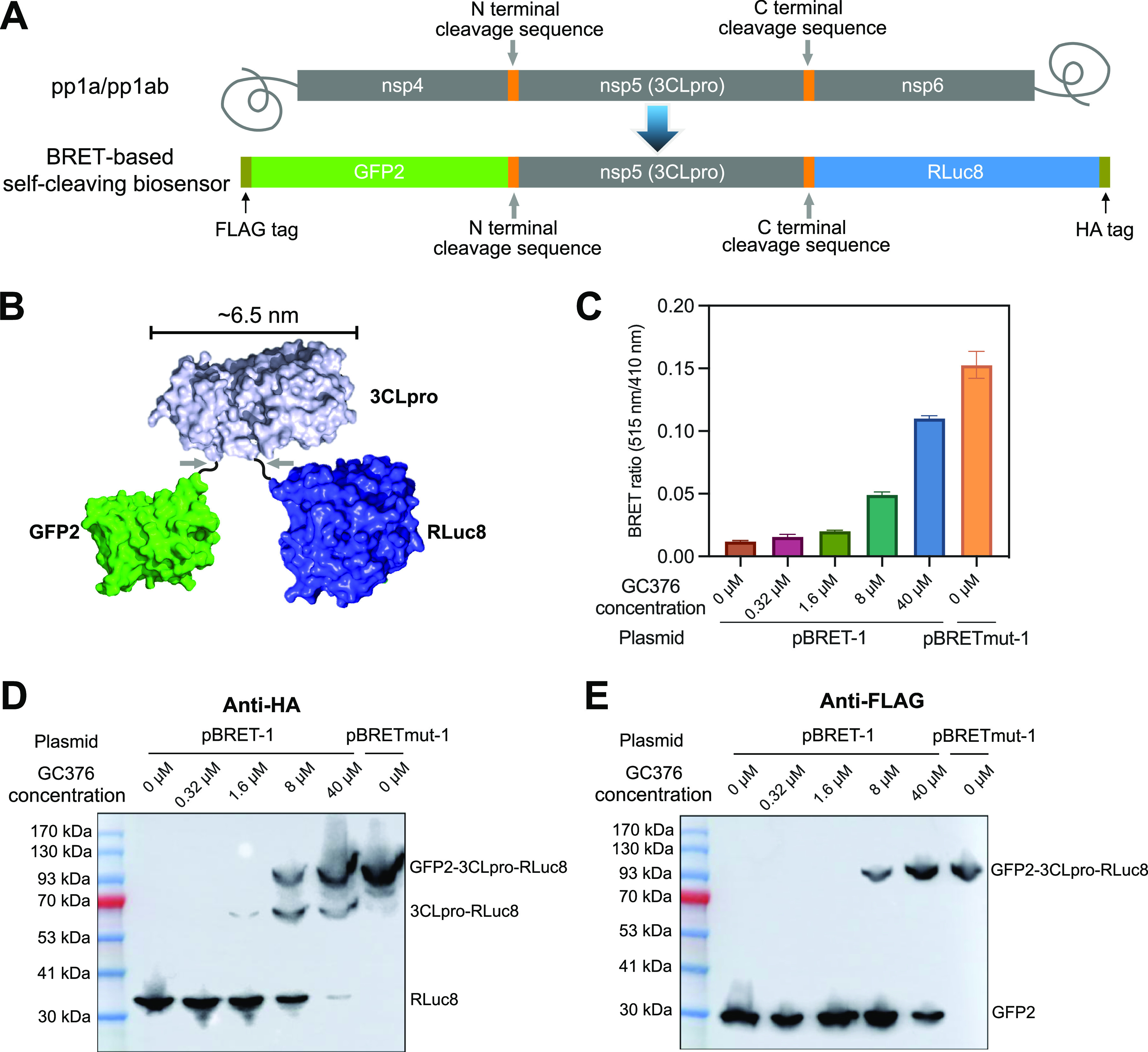
(A) The domain organization of SARS-CoV-2 polyproteins (pp1a or pp1ab) (top) and the BRET-based self-cleaving biosensor (bottom). (B) An illustration of the BRET-based self-cleaving biosensor, showing the relative positions of GFP2, 3Clpro, and RLuc8. (C) The BRET ratio of HEK 293T cells 24 h post-transfection of the plasmid carrying biosensor pBRET-1. The 3CLpro inhibitor GC376, at the indicated working concentrations, was added right after transfection. The biosensor with a C145A mutation in 3CLpro (pBRETmut-1) was used as a noncleavable control. The data represent the mean ± standard deviation of three independent measurements. (D and E) The self-cleavage of pBRET-1 in the presence of the indicated concentrations of GC376 was detected by Western blotting using an anti-HA antibody (D) or an anti-FLAG antibody (E).

In this study, we develop a series of BRET-based biosensors to set up cell-based assays for 3CLpro inhibitor discovery. We linked a green fluorescent protein (GFP2) and a Renilla luciferase (RLuc8) to the N- and C-terminus of SARS-CoV-2 3CLpro, respectively, using 3CLpro cleavage sequences as the linkers (Fig. 1A and B). RLuc8 can catalyze the oxidation of its substrate, coelenterazine 400a, by molecular oxygen (O_2_) to produce light with a peak wavelength at 395 nm. This light can be absorbed by GFP2 in the biosensors to emit light with a peak wavelength at 510 nm. The energy transfer from RLuc8 to GFP2 can be disrupted by self-cleavage catalyzed by 3CLpro and restored by adding 3CLpro inhibitors. We use RLuc8 as the BRET donor and GFP2 as the BRET acceptor because their emission peaks are well separated, and they can give a better BRET signal (the emission at 510 nm) in comparison with other BRET pairs, such as RLuc and enhanced YFP (19). The effect of the variance in the biosensor expression levels on the readouts was minimized by normalizing the BRET signal with the luminescent signal of RLuc8.

## RESULTS AND DISCUSSION

### Design of a BRET-based self-cleaving biosensor, pBRET-1.

The maximal distance for BRET is about 10 nm (19). According to a crystal structure of SARS-CoV-2 3CLpro (PDB code: 6Y2E), the distance between the N- and C-terminus of 3CLpro is 22.9 Å (11). To construct our first biosensor, pBRET-1, we linked GFP2 to the N-terminus of SARS-CoV-2 3CLpro, using the cleavage sequence between nsp4 and 3CLpro as the linker, and linked RLuc8 to the C-terminus of 3CLpro, using the cleavage sequence between 3CLpro and nsp6 as the linker. Further, we added a FLAG tag before GFP2 and a HA tag after RLuc8 (Fig. 1A). We also constructed pBRETmut-1, in which the catalytic residue C145 of 3CLpro was mutated to alanine.

The transient expression of pBRET-1 in HEK 293T cells resulted in a BRET ratio (see Materials and Methods) of about 0.01, while the transient expression of pBRETmut-1 showed a BRET ratio of 0.15 (Fig. 1C). Adding GC376, a reported inhibitor of 3CLpro (8), to the cell culture increased the BRET ratio of pBRET-1 in a concentration-dependent manner. These results indicate that the BRET ratio of pBRET-1 is negatively associated with the protease activity of 3CLpro.

We also monitored the self-cleavage of pBRET-1 in HEK 293T cells using Western blot. We first used an anti-HA antibody to detect the self-cleavage products (Fig. 1D). For pBRET-1 in the absence of GC376, only RLuc was detected. As the concentration of GC376 increased, the band of the 3CLpro-RLuc8 fragment and that of the full-length GFP2-3CLpro-Rluc8 fusion protein appeared. We also used an anti-FLAG antibody to detect the self-cleavage products (Fig. 1E). Interestingly, as the concentration of GC376 increased, only the bands of GFP2 and the full-length GFP2-3CLpro-Rluc8 fusion protein were detected, with no appearance of the band of the GFP2-3CLpro fragment. These results suggest that the cleavage at the N-terminus of 3CLpro occurred before the cleavage at the C-terminus, which is consistent with the previously reported maturation process of 3CLpro (20).

### Optimization of pBRET-1.

**FIG 2:**
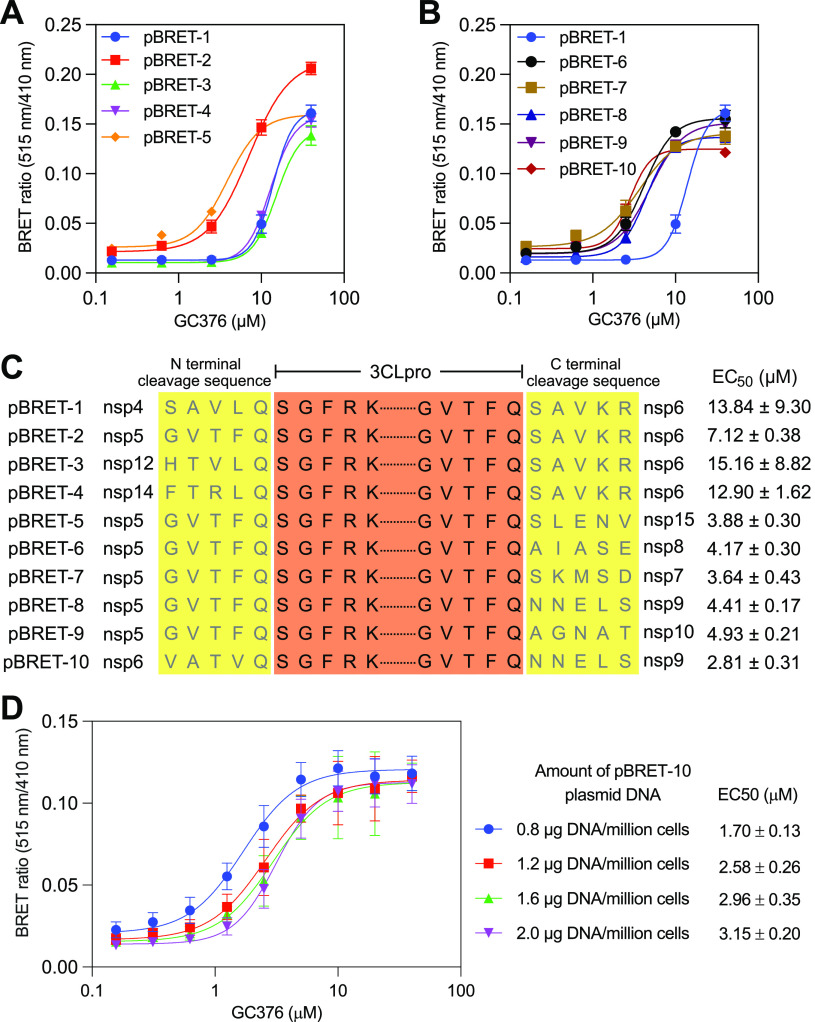
(A and B) The BRET ratio of HEK 293T cells 24 h post-transfection of the different biosensor plasmids (pBRET-1 to pBRET-10). The 3CLpro inhibitor GC376 was diluted into cell culture media at the indicated working concentrations right after transfection. The data represent the mean ± standard deviation of three independent measurements. (C) The amino acid sequences of the 3CLpro cleavage sites at the N- and C-terminus of the 10 biosensors, as well as the EC_50_ of GC376 measured using these biosensors. (D) The EC_50_ values of GC376 measured in HEK 293T cells transfected with different amounts of pBRET-10 plasmid DNA. The data represent the mean ± standard deviation of five independent measurements.

There are 11 3CLpro cleavage sites in the polyproteins pp1a and pp1ab of SARS-CoV-2 (21). A study of the substrate specificity of SARS-CoV 3CLpro showed that the efficiencies of 3CLpro to cleave its substrates were highly dependent on the substrate sequences (22). We presume that modulating the self-cleaving efficiency of pBRET-1 by changing the cleavage sequence between GFP2 and 3CLpro as well as that between 3CLpro and RLuc8 may increase the sensitivity of the biosensor to 3CLpro inhibitors. As shown in Fig. 2, nine pBRET biosensors were designed, and their sensitivities to GC376 were tested. Among them, pBRET-10 showed the highest sensitivity, with an EC_50_ value of 2.81 μM for GC376. In contrast, the EC_50_ value measured using pBRET-1 was 13.84 μM (Fig. 2C).

To test the robustness of our biosensor-based assay, a noncleavable version of pBRET-10 was constructed by introducing a 3CLpro C145A mutation (named pBRETmut-10). Different amounts of this plasmid were transferred to HEK 293T cells. The luciferase signal, which represented the expression level of pBRETmut-10, almost tripled when the plasmid amount was increased from 0.8 to 2.0 μg/million cells. In contrast, the BRET ratio only slightly changed from 0.12 to 0.13 (Fig. S1). Next, the activity of GC376 in HEK 293T cells was measured using different amounts of pBRET-10 plasmid DNA. The EC_50_ value of GC376 was increased from 1.70 μM to 3.15 μM when the amount of pBRET-10 plasmid DNA was increased from 0.8 to 2.0 μg/million cells (Fig. 2D). The data indicate that the expression level of the biosensor has a small effect on the readouts (the BRET ratios) but that the calculated EC_50_ value has a positive correlation with the biosensor expression level, probably because a higher concentration of 3CLpro requires a higher concentration of the inhibitor to inhibit it.

### Evaluation of the cellular activities of 3CLpro inhibitors using pBRET-10.

As pBRET-10 has the highest sensitivity, we measured the EC_50_ values of three other 3CLpro inhibitors (Boceprevir and compounds 11a and 13b) using pBRET-10 (Table 1) (5, 8, 11). Compound 11a showed an activity slightly lower than that of GC376, while the activities of Boceprevir and compound 13b were an order of magnitude lower than that of GC376. The EC_50_ values of GC376 and Boceprevir are comparable to that measured using cell-based anti-SARS-CoV-2 assays (8), but for compounds 11a and 13b, the EC_50_ values from our measurements were about 9 times the reported values from anti-SARS-CoV-2 assays (5, 11).

**TABLE 1:** The EC_50_ values of four reported 3CLpro inhibitors measured using pBRET-10 (1.6 μg plasmid DNA/million cells) and the corresponding EC_50_ values from anti-SARS-CoV-2 assays

3CLpro inhibitors	EC_50_ measured using pBRET-10 (μM)	Reported EC_50_ against SARS-CoV-2 (μM)	References
GC376	3.45 ± 0.16	0.70 (Vero E6 cells, MOI 0.01); 2.20 (Vero E6 cells, MOI 0.01)	(8, 14)
Boceprevir	36.45 ± 0.61	15.57 (Vero E6 cells, MOI 0.01)	(8)
11a	4.96 ± 0.38	0.53 (Vero E6 cells, MOI 0.05)	(5)
13b	37.03 ± 0.64	4 to 5 (Calu-3 cells, MOI 0.05)	(11)

### Conclusion.

We have developed a class of BRET-based self-cleaving biosensors that can be used in BSL-2 laboratories to set up cell-based assays for 3CLpro inhibitor discovery. One of them, pBRET-10, showed comparable sensitivity to cell-based antiviral assays. Self-cleavage catalyzed by 3CLpro in these biosensors mimics the activation process of 3CLpro during coronavirus replication. In addition to the 3CLpro of SARS-CoV-2, similar biosensors can be developed to screen the inhibitors of the 3C-like proteases of other coronaviruses. Furthermore, many other viruses (such as HIV, HCV, Dengue virus, Zika virus, and West Nile virus) also utilize a replication strategy involving the expression of a polyprotein containing a self-cleaving protease (23–27). Therefore, our strategy can also be used to develop biosensors for proteases of these viruses, such as the HIV protease PR and HCV protease NS3.

## MATERIALS AND METHODS

### Construction of plasmids.

The gene sequences of RLuc8 and GFP2 are the same as those reported previously (28). The gene sequences of SARS-CoV-2 3CLpro and its cleavage sites are the same as those in the SARS-CoV-2 genome (NC_045512.2). The DNA fragment encoding the biosensor pBRET-1 was synthesized at GENEWIZ (Suzhou, China) and inserted into the pcDNA3.1 vector at the site after the FLAG-tag. Then, an HA-tag was added to the C-terminus of pBRET-1 via Gibson homologous recombination using primers HA-F and HA-R (Table S1). The plasmid of pBRETmut-1 was constructed by introducing the 3CLpro C145A mutation into pBRET-1 through site-directed mutagenesis using primers C145A-F and C145A-R (Table S1). The plasmids carrying other pBRET biosensors were constructed on the basis of pBRET-1 using the Gibson homologous recombination method. The primers are shown in Table S1. The protein sequences of all of the BRET-based self-cleaving biosensors are shown in Table S2.

### Cell culture.

HEK 293T cells were cultured in a humidified incubator maintained at 37°C with 5% CO_2_, using the high-glucose Dulbecco’s modified Eagle’s medium (Gibco) supplemented with 100 U/mL penicillium-streptomycin (HyClone) and 10% fetal bovine serum (Gibco).

### 3CLpro inhibitors.

GC376 (Selleck, S0475) and Boceprevir (Selleck, S3733) were purchased from Selleck. Compounds 11a and 13b were synthesized following previously reported protocols (5, 11).

### Western blot and BRET assays.

To monitor the self-cleavage of pBRET-1 and to evaluate its sensitivity to 3CLpro inhibitor GC376, HEK 293T cells were seeded into 6-well cell culture plates at approximately 40% confluence, and after 24 h, the cells were transfected with plasmids carrying pBRET-1 (5 μg/well, approximately 0.8 μg/million cells), using PEI as the transfection reagent. After transfection, 3CLpro inhibitors in DMSO were added into the cell culture to reach the indicated working concentrations. The final DMSO concentration in the cell culture was 0.5%. After an additional 24 h, the medium was removed. The cells in each well, were washed twice with ice-cold PBS buffer and then resuspended in 1 mL ice-cold PBS buffer. Next, 900 μL were used for the Western blot, and 100 μL were used for the BRET assay.

For the Western blot, the PBS buffer was removed by centrifugation, and the HEK 293T cells were lysed using 100 μL of RIPA buffer (Beyotime, P0013B). Then, equal amounts of total protein under each condition were resolved by SDS-PAGE and transferred to a PVDF membrane (Merck Millipore, ISEQ00010). The PVDF membrane was blocked with 1% (for anti-FLAG antibody) or 3% (for anti-HA antibody) nonfat milk in TBST buffer (Tris-buffered saline containing 0.05% Tween 20) overnight at 4°C and incubated with an anti-FLAG antibody (Sigma, F1804, 1:1000 dilution) or an anti-HA antibody (Abcam, ab9110, 1:1000 dilution) for 2 h at room temperature. After being washed three times with TBST buffer, the PVDF membrane was incubated with anti-rabbit IgG (Merck Millipore, AP156P, 1:20000 dilution) or anti-mouse IgG (Merck Millipore, AP127P; 1:20000 dilution) for 1 h, washed with TBST buffer three times, and developed using Pierce ECL Western blotting substrate (CWBIO, CW0049S).

For the BRET assay, the cells were resuspended in ice-cold PBS and transferred to a 96-well clear-bottom white plate (Corning, 3610). After adding coelenterazine 400a (Cayman, 16157) to a final concentration of 20 μM, the luminous signal at 410 nm and the fluorescent signal at 515 nm were measured using a BioTek microplate reader (Biotek Synergy NEO2). The BRET ratio was calculated using the following equation:

$$\text{BRET ratio }=\;\left({\text{F}}_{\text{515},\text{S}}-{\text{F}}_{\text{515},\text{BL}}\right)/\left({\text{L}}_{\text{41}0,\text{S}}-{\text{L}}_{\text{41}0,\text{BL}}\right),$$

in which F_515,S_ and L_410,S_ are the fluorescent (515 nm) and luminescent (410 nm) signals, respectively, of cells expressing pBRET biosensors, and F_515,BL_ and L_410,BL_ are the fluorescent (515 nm) and luminescent (410 nm) signals, respectively, of HEK 293T cells without pBRET biosensors.

To set up the high-throughput BRET assay for the optimization of pBRET-1 and evaluation of the activities of the different 3CLpro inhibitors, HEK 293T cells were seeded into a 96-well clear-bottom white plate (Corning, 3610) at approximately 40% confluence. After 24 h, the cells were transfected with plasmids carrying the biosensors (0.4 μg/well, about 1.6 μg/million cells) using PEI as the transfection reagent. Then, 3CLpro inhibitors in DMSO were added into the cell culture to reach the indicated working concentrations. Twenty-four hours later, the BRET ratios were measured using the same protocols as described above.
