# Supplementary material for: BRET-Based Self-Cleaving Biosensors for SARS-CoV-2 3CLpro Inhibitor Discovery
Source: Microbiol Spectr. 2022 Jun 27;10(4):e02559-21. doi: 10.1128/spectrum.02559-21 (PMC9430692; doi:10.1128/spectrum.02559-21)
Supplement: Supplemental file 1 — Fig. S1; Tables S1 to S7. Download spectrum.02559-21-s0001.pdf, PDF file, 0.5 MB [file spectrum.02559-21-s0001.pdf]

Supporting Information

Title: BRET-based self-cleaving biosensors for SARS-CoV-2 3CLpro Inhibitor Discovery

Author names: Ningke Hou[a,b,c], Chen Peng[a,b,c], Lijing Zhang[a,b,c], Yuyao Zhu[a,b,c], Qi Hu[a,b,c]*

Affiliations:

[a]Key Laboratory of Structural Biology of Zhejiang Province, School of Life Sciences, Westlake University, Hangzhou, Zhejiang, China.

[b]Center for Infectious Disease Research, Westlake Laboratory of Life Sciences and Biomedicine, Hangzhou, Zhejiang, China.

[c]Institute of Biology, Westlake Institute for Advanced Study, Hangzhou, Zhejiang, China.

***Corresponding author:** Email: huqi@westlake.edu.cn; Tel: (86)0571-85273915

This supporting information file contains:

Figure S1. The luciferase signal (410 nm) and the BRET ratio of pBRETmut-10 transiently expressed in HEK 293T cells.

Table S1. The primer sequences used in construction of plasmids of BRET-based self-cleaving biosensors.

Table S2. The protein sequences of BRET-based self-cleaving biosensors.

Table S3. Experimental data of Figure 1C.

Table S4. Experimental data of Figures 2A, 2B and 2C.

Table S5. Experimental data of Figure 2D.

Table S6. Experimental data of Table 1.

Table S7. Experimental data of Figure S1.

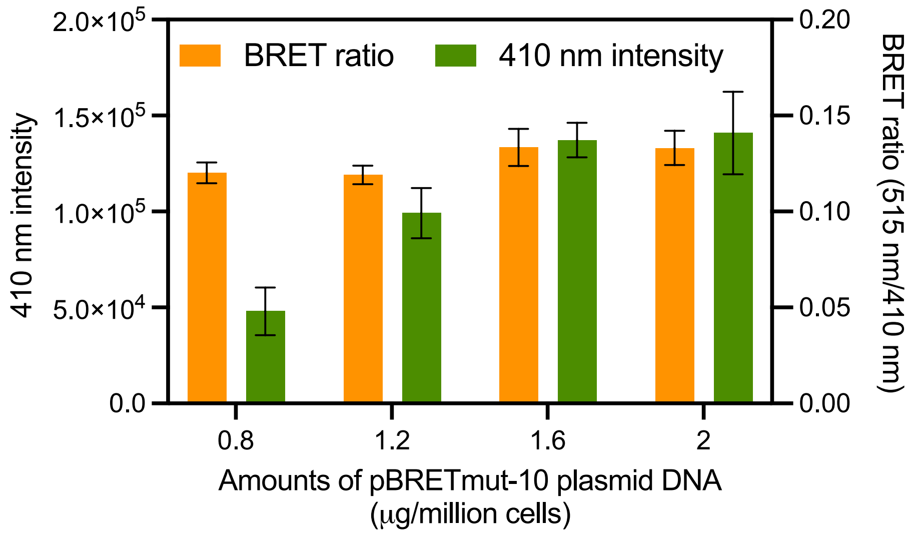

Figure S1. The luciferase signal (410 nm) and the BRET ratio of pBRETmut-10 transiently expressed in HEK 293T cells. The data represent the mean ± SD of four independent measurements.

Table S1. The primer sequences used in construction of plasmids of BRET-based self-cleaving biosensors.

Primer name	Sequences
HA-F	5’-ACCCATACGATGTTCCAGATTACGCTTAATCTAGAGGGCCCGTTTAAACCCG-3’
HA-R	5’-TGGAACATCGTATGGGTAAGATCCCTCGAGCTGCTCGTTCTTCAGCAC-3’
C145A-F	5’-GGTTCAGCTGGTAGTGTTGGTTTTAACATAGATTATGACTGTGTCTC-3’
C145A-R	5’-ACACTACCAGCTGAACCATTAAGGAATGAACCCTTAATAGTGAAATTG-3’
pBRET-2-F	5’-GGTGTTACTTTCCAAAGTGGTTTTAGAAAAATGGCATTCCCATCTGG-3’
pBRRT-2-R	TTGGAAAGTAACACCAGATCCTCCGCCGCCCTTG-3’
pBRET-3-F	CATACAGTCTTACAGAGTGGTTTTAGAAAAATGGCATTCCCATCTGG-3’
pBRET-3-R	CTGTAAGACTGTATGAGATCCTCCGCCGCCCTTG-3’
pBRET-4-F	TTTACAAGACTTCAGAGTGGTTTTAGAAAAATGGCATTCCCATCTGG-3’
pBRET-4-R	CTGAAGTCTTGTAAAAGATCCTCCGCCGCCCTTG-3’
pBRET-5-F	AGTTTAGAAAATGTGGGCGGCGGAGGATCTATG-3’
pBRET-5-R	CACATTTTCTAAACTTTGGAAAGTAACACCTGAGCATTGTC-3’
pBRET-6-F	GCTATAGCCTCAGAGGGCGGCGGAGGATCTATGAC-3’
pBRET-6-R	CTCTGAGGCTATAGCTTGGAAAGTAACACCTGAGCATTGTCTAAC-3’
pBRET-7-F	TCTAAAATGTCAGATGGCGGCGGAGGATCTATGAC-3’
pBRET-7-R	ATCTGACATTTTAGATTGGAAAGTAACACCTGAGCATTGTCTAAC-3’
pBRET-8-F	AATAATGAGCTTAGTGGCGGCGGAGGATCTATGAC-3’
pBRET-8-R	ACTAAGCTCATTATTTGGAAAGTAACACCTGAGCATTGTCTAAC-3’
pBRET-9-F	GCTGGTAATGCAACAGGCGGCGGAGGATCTATGAC-3’
pBRET-9-R	TGTTGCATTACCAGCTTGGAAAGTAACACCTGAGCATTGTCTAAC-3’
pBRET-10-F	GTAGCCACTGTACAGAGTGGTTTTAGAAAAATGGCATTCCCATCTGG-3’
pBRET-10-R	CTGTACAGTGGCTACAGATCCTCCGCCGCCCTTG-3’

Table S2. The protein sequences of BRET-based self-cleaving biosensors.

pBRET biosensors	Protein sequences[1]
pBRET-1	MDYKDDDDKMVSKGEELFTGVVPILVELDGDVNGHKFSVSGEGEGD ATYGKLTLKFICTTGKLPVPWPTLVTTLSYGVQCFSRYPDHMKQHDFF KSAMPEGYVQERTIFFKDDGNYKTRAEVKFEGDTLVNRIELKGIDFKE DGNILGHKLEYNYNSHNVYIMADKQKNGIKVNFKIRHNIEDGSVQLA DHYQQNTPIGDGPVLLPDNHYLSTQSALSKDPNEKRDHMVLLEFVTA AGITLGMDELYKGGGGSSAVLQSGFRKMAFPSGKVEGCMVQVTCGTT TLNGLWLDDVVYCPRHVICTSEDMLNPNYEDLLIRKSNHNFLVQAGN VQLRVIGHSMQNCVLKLKVDTANPKTPKYKFVRIQPGQTFSVLACYN GSPSGVYQCAMRPNFTIKGSFLNGSCGSVGFNIDYDCVSFCYMHHME LPTGVHAGTDLEGNFYGPFVDRQTAQAAGTDTTITVNVLAWLYAAVIN GDRWFLNRFTTTLNDFNLVAMKYNYEPLTQDHVDILGPLSAQTGIAVL DMCASLKELLQNGMNGRTILGSALLEDEFTPFDVVRQCSGVTFQSAVK RGGGGSMTSKVYDPEQRKRMITGPQWWARCKQMNVLDSFINYYDSE KHAENAVIFLHGNATSSYLWRHVVPHIEPVARCIIPDLIGMGKSGKSGN GSYRLLDHYKYLTAWFELLNLPKKIIFVGHDWGAALAFHYAYEHQDRI KAIVHMESVVDVIESWDEWPDIEEDIALIKSEEGEKMVLENNFFVETV LPSKIMRKLEPEEFAAYLEPFKEKGEVRRPTLSWPREIPLVKGGKPDVV QIVRNYNAYLRASDDLPKLFIESDPGFFSNAIVEGAKKFPNTEFVKVKG LHFLQEDAPDEMGKYIKSFVERVLKNEQLEGSYPYDVPDYA*
pBRET-2	MDYKDDDDKMVSKGEELFTGVVPILVELDGDVNGHKFSVSGEGEGD ATYGKLTLKFICTTGKLPVPWPTLVTTLSYGVQCFSRYPDHMKQHDFF KSAMPEGYVQERTIFFKDDGNYKTRAEVKFEGDTLVNRIELKGIDFKE DGNILGHKLEYNYNSHNVYIMADKQKNGIKVNFKIRHNIEDGSVQLA DHYQQNTPIGDGPVLLPDNHYLSTQSALSKDPNEKRDHMVLLEFVTA AGITLGMDELYKGGGGSGVTFQSGFRKMAFPSGKVEGCMVQVTCGT TTLNGLWLDDVVYCPRHVICTSEDMLNPNYEDLLIRKSNHNFLVQAG NVQLRVIGHSMQNCVLKLKVDTANPKTPKYKFVRIQPGQTFSVLACY

	NGSPSGVYQCAMRPNFTIKGSFLNGSCGSVGFNIDYDCVSFCYMHHM ELPTGVHAGTDLEGNFYGPFVDRQTAQAAGTDTTITVNVLAWLYAAVI NGDRWFLNRFTTTLNDFNLVAMKYNYEPLTQDHVDILGPLSAQTGIAV LDMCASLKELLQNGMNGRTILGSALLEDEFTPFDVVRQCSGVTFQSAV KRGGGGSMTSKVYDPEQRKRMITGPQWWARCKQMNVLDSFINYYDS EKHAENAVIFLHGNATSSYLWRHVVPHIEPVARCIIPDLIGMGKSGKSG NGSYRLLDHYKYLTAWFELLNLPKKIIFVGHDWGAALAFHYAYEHQD RIKAIVHMESVVDVIESWDEWPDIEEDIALIKSEEGEKMVLENNFFVET VLPSKIMRKLEPEEFAAYLEPFKEKGEVRRPTLSWPREIPLVKGGKPDV VQIVRNYNAYLRASDDLPKLFIESDPGFFSNAIVEGAKKFPNTEFVKVK GLHFLQEDAPDEMGKYIKSFVERVLKNEQLEGSYPYDVPDYA*
pBRET-3	MDYKDDDDKMVSKGEELFTGVVPILVELDGDVNGHKFSVSGEGEGD ATYGKLTLKFICTTGKLPVPWPTLVTTLSYGVQCFSRYPDHMKQHDFF KSAMPEGYVQERTIFFKDDGNYKTRAEVKFEGDTLVNRIELKGIDFKE DGNILGHKLEYNYNSHNVYIMADKQKNGIKVNFKIRHNIEDGSVQLA DHYQQNTPIGDGPVLLPDNHYLSTQSALSKDPNEKRDHMVLLEFVTA AGITLGMDELYKGGGGSHTVLQSGFRKMAFPSGKVEGCMVQVTCGT TTLNGLWLDDVVYCPRHVICTSEDMLNPNYEDLLIRKSNHNFLVQAG NVQLRVIGHSMQNCVLKLKVDTANPKTPKYKFVRIQPGQTFSVLACY NGSPSGVYQCAMRPNFTIKGSFLNGSCGSVGFNIDYDCVSFCYMHHM ELPTGVHAGTDLEGNFYGPFVDRQTAQAAGTDTTITVNVLAWLYAAVI NGDRWFLNRFTTTLNDFNLVAMKYNYEPLTQDHVDILGPLSAQTGIAV LDMCASLKELLQNGMNGRTILGSALLEDEFTPFDVVRQCSGVTFQSAV KRGGGGSMTSKVYDPEQRKRMITGPQWWARCKQMNVLDSFINYYDS EKHAENAVIFLHGNATSSYLWRHVVPHIEPVARCIIPDLIGMGKSGKSG NGSYRLLDHYKYLTAWFELLNLPKKIIFVGHDWGAALAFHYAYEHQD RIKAIVHMESVVDVIESWDEWPDIEEDIALIKSEEGEKMVLENNFFVET VLPSKIMRKLEPEEFAAYLEPFKEKGEVRRPTLSWPREIPLVKGGKPDV VQIVRNYNAYLRASDDLPKLFIESDPGFFSNAIVEGAKKFPNTEFVKVK

	GLHFLQEDAPDEMGKYIKSFVERVLKNEQLEGSYPYDVPDYA*
pBRET-4	MDYKDDDDKMVSKGEELFTGVVPILVELDGDVNGHKFSVSGEGEGD<br>ATYGKLTLKFICTTGKLPVPWPTLVTTLSYGVQCFSRYPDHMKQHDFF<br>KSAMPEGYVQERTIFFKDDGNYKTRAEVKFEGDTLVNRIELKGIDFKE<br>DGNILGHKLEYNYNSHNVYIMADKQKNGIKVNFKIRHNIEDGSVQLA<br>DHYQQNTPIGDGPVLLPDNHYLSTQSALSKDPNEKRDHMVLLEFVTA<br>AGITLGMDELYKGGGGSFTRLQSGFRKMAFPSGKVEGCMVQVTCGTT<br>TLNGLWLDDVVYCPRHVICTSEDMLNPNYEDLLIRKSNHNFLVQAGN<br>VQLRVIGHSMQNCVLKLKVDTANPKTPKYKFVRIQPGQTFSVLACYN<br>GSPSGVYQCAMRPNFTIKGSFLNGSCGSVGFNIDYDCVSFCYMHHME<br>LPTGVHAGTDLEGNFYGPFVDRQTAQAAGTDTTITVNVLAWLYAAVIN<br>GDRWFLNRFTTTLNDFNLVAMKYNYEPLTQDHVDILGPLSAQTGIAVL<br>DMCASLKELLQNGMNGRTILGSALLEDEFTPFDVVRQCSGVTFQSAVK<br>RGGGGSMTSKVYDPEQRKRMITGPQWWARCKQMNVLDSFINYYDSE<br>KHAENAVIFLHGNATSSYLWRHVVPHIEPVARCIIPDLIGMGKSGKSGN<br>GSYRLLDHYKYLTAWFELLNLPKKIIFVGHDWGAALAFHYAYEHQDRI<br>KAIVHMESVVDVIESWDEWPDIEEDIALIKSEEGEKMVLENNFFVETV<br>LPSKIMRKLEPEEFAAYLEPFKEKGEVRRPTLSWPREIPLVKGGKPDVV<br>QIVRNYNAYLRASDDLPKLFIESDPGFFSNAIVEGAKKFPNTEFVKVKG<br>LHFLQEDAPDEMGKYIKSFVERVLKNEQLEGSYPYDVPDYA*
pBRET-5	MDYKDDDDKMVSKGEELFTGVVPILVELDGDVNGHKFSVSGEGEGD<br>ATYGKLTLKFICTTGKLPVPWPTLVTTLSYGVQCFSRYPDHMKQHDFF<br>KSAMPEGYVQERTIFFKDDGNYKTRAEVKFEGDTLVNRIELKGIDFKE<br>DGNILGHKLEYNYNSHNVYIMADKQKNGIKVNFKIRHNIEDGSVQLA<br>DHYQQNTPIGDGPVLLPDNHYLSTQSALSKDPNEKRDHMVLLEFVTA<br>AGITLGMDELYKGGGGSGVTFQSGFRKMAFPSGKVEGCMVQVTCGT<br>TTLNGLWLDDVVYCPRHVICTSEDMLNPNYEDLLIRKSNHNFLVQAG<br>NVQLRVIGHSMQNCVLKLKVDTANPKTPKYKFVRIQPGQTFSVLACY<br>NGSPSGVYQCAMRPNFTIKGSFLNGSCGSVGFNIDYDCVSFCYMHHM

	ELPTGVHAGTDLEGNFYGPFVDRQTAQAAGTDTTITVNVLAWLYAAVINGDRWFLNRFTTTLNDFNLVAMKYNYEPLTQDHVDILGPLSAQTGIAVLDMCASLKELLQNGMNGRTILGSALLEDEFTPFDVVRQCSGVTFQSLENVGGGGSMTSKVYDPEQRKRMITGPQWWARCKQMNVLDSFINYYDSEKHAENAVIFLHGNATSSYLWRHVVPHIEPVARCIIPDLIGMGKSGKSGNGSYRLLDHYKYLTAWFELLNLPKKIIFVGHDWGAALAFHYAYEHQDRIKAIVHMESVVDVIESWDEWPDIEEDIALIKSEEGEKMVLENNFFVETVLPSKIMRKLEPEEFAAYLEPFKEKGEVRRPTLSWPREIPLVKGGKPDVVQIVRNYNAYLRASDDLPKLFIESDPGFFSNAIVEGAKKFPNTEFVKVKGLHFLQEDAPDEMGKYIKSFVERVLKNEQLEGSYPYDVPDYA*
pBRET-6	MDYKDDDDKMVSKGEELFTGVVPILVELDGDVNGHKFSVSGEGEGDATYGKLTLKFICTTGKLPVPWPTLVTTLSYGVQCFSRYPDHMKQHDFFKSAMPEGYVQERTIFFKDDGNYKTRAEVKFEGDTLVNRIELKGIDFKEDGNILGHKLEYNYNSHNVYIMADKQKNGIKVNFKIRHNIEDGSVQLADHYQQNTPIGDGPVLLPDNHYLSTQSALSKDPNEKRDHMVLLEFVTAAGITLGMDELYKGGGGSGVTFQSGFRKMAFPSGKVEGCMVQVTCGTTTLNGLWLDDVVYCPRHVICTSEDMLNPNYEDLLIRKSNHNFLVQAGNVQLRVIGHSMQNCVLKLKVDTANPKTPKYKFVRIQPGQTFSVLACYNGSPSGVYQCAMRPNFTIKGSFLNGSCGSVGFNIDYDCVSFCYMHHMELPTGVHAGTDLEGNFYGPFVDRQTAQAAGTDTTITVNVLAWLYAAVINGDRWFLNRFTTTLNDFNLVAMKYNYEPLTQDHVDILGPLSAQTGIAVLDMCASLKELLQNGMNGRTILGSALLEDEFTPFDVVRQCSGVTFQAIASEGGGGSMTSKVYDPEQRKRMITGPQWWARCKQMNVLDSFINYYDSEKHAENAVIFLHGNATSSYLWRHVVPHIEPVARCIIPDLIGMGKSGKSGNGSYRLLDHYKYLTAWFELLNLPKKIIFVGHDWGAALAFHYAYEHQDRIKAIVHMESVVDVIESWDEWPDIEEDIALIKSEEGEKMVLENNFFVETVLPSKIMRKLEPEEFAAYLEPFKEKGEVRRPTLSWPREIPLVKGGKPDVVQIVRNYNAYLRASDDLPKLFIESDPGFFSNAIVEGAKKFPNTEFVKVKGLHFLQEDAPDEMGKYIKSFVERVLKNEQLEGSYPYDVPDYA*

pBRET-7	MDYKDDDDKMVSKGEELFTGVVPILVELDGDVNGHKFSVSGEGEGD ATYGKLTLKFICTTGKLPVPWPTLVTTLSYGVQCFSRYPDHMKQHDFF KSAMPEGYVQERTIFFKDDGNYKTRAEVKFEGDTLVNRIELKGIDFKE DGNILGHKLEYNYNSHNVYIMADKQKNGIKVNFKIRHNIEDGSVQLA DHYQQNTPIGDGPVLLPDNHYLSTQSALSKDPNEKRDHMVLLEFVTA AGITLGMDELYKGGGGSGVTFQSGFRKMAFPSGKVEGCMVQVTCGT TTLNGLWLDDVVYCPRHVICTSEDMLNPNYEDLLIRKSNHNFLVQAG NVQLRVIGHSMQNCVLKLKVDTANPKTPKYKFVRIQPGQTFSVLACY NGSPSGVYQCAMRPNFTIKGSFLNGSCGSVGFNIDYDCVSFCYMHHM ELPTGVHAGTDLEGNFYGPFVDRQTAQAAGTDTTITVNVLAWLYAAVI NGDRWFLNRFTTTLNDFNLVAMKYNYEPLTQDHVDILGPLSAQTGIAV LDMCASLKELLQNGMNGRTILGSALLEDEFTPFDVVRQCSGVTFQSK MSDGGGGSMTSKVYDPEQRKRMITGPQWWARCKQMNVLDSFINYY DSEKHAENAVIFLHGNATSSYLWRHVVPHIEPVARCIIPDLIGMGKSGK SGNGSYRLLDHYKYLTAWFELLNLPKKIIFVGHDWGAALAFHYAYEH QDRIKAIVHMESVVDVIESWDEWPDIEEDIALIKSEEGEKMVLENNFFV ETVLPSKIMRKLEPEEFAAYLEPFKEKGEVRRPTLSWPREIPLVKGGKP DVVQIVRNYNAYLRASDDLPKLFIESDPGFFSNAIVEGAKKFPNTEFVK VKGLHFLQEDAPDEMGKYIKSFVERVLKNEQLEGSYPYDVPDYA*
pBRET-8	MDYKDDDDKMVSKGEELFTGVVPILVELDGDVNGHKFSVSGEGEGD ATYGKLTLKFICTTGKLPVPWPTLVTTLSYGVQCFSRYPDHMKQHDFF KSAMPEGYVQERTIFFKDDGNYKTRAEVKFEGDTLVNRIELKGIDFKE DGNILGHKLEYNYNSHNVYIMADKQKNGIKVNFKIRHNIEDGSVQLA DHYQQNTPIGDGPVLLPDNHYLSTQSALSKDPNEKRDHMVLLEFVTA AGITLGMDELYKGGGGSGVTFQSGFRKMAFPSGKVEGCMVQVTCGT TTLNGLWLDDVVYCPRHVICTSEDMLNPNYEDLLIRKSNHNFLVQAG NVQLRVIGHSMQNCVLKLKVDTANPKTPKYKFVRIQPGQTFSVLACY NGSPSGVYQCAMRPNFTIKGSFLNGSCGSVGFNIDYDCVSFCYMHHM ELPTGVHAGTDLEGNFYGPFVDRQTAQAAGTDTTITVNVLAWLYAAVI

	NGDRWFLNRFTTTLNDFNLVAMKYNYEPLTQDHVDILGPLSAQTGIAV LDMCASLKELLQNGMNGRTILGSALLEDEFTPFDVVRQCSGVTFQNN ELSGGGGSMTSKVYDPEQRKRMITGPQWWARCKQMNVLDSFINYYD SEKHAENAVIFLHGNATSSYLWRHVVPHIEPVARCIIPDLIGMGKSGKS GNGSYRLLDHYKYLTAWFELLNLPKKIIFVGHDWGAALAFHYAYEHQ DRIKAIVHMESVVDVIESWDEWPDIEEDIALIKSEEGEKMVLENNFFVE TVLPSKIMRKLEPEEFAAYLEPFKEKGEVRRPTLSWPREIPLVKGGKPD VVQIVRNYNAYLRASDDLPKLFIESDPGFFSNAIVEGAKKFPNTEFVKV KGLHFLQEDAPDEMGKYIKSFVERVLKNEQLEGSYPYDVPDYA*
pBRET-9	MDYKDDDDKMVSKGEELFTGVVPILVELDGDVNGHKFSVSGEGEGD ATYGKLTLKFICTTGKLPVPWPTLVTTLSYGVQCFSRYPDHMKQHDFF KSAMPEGYVQERTIFFKDDGNYKTRAEVKFEGDTLVNRIELKGIDFKE DGNILGHKLEYNYNSHNVYIMADKQKNGIKVNFKIRHNIEDGSVQLA DHYQQNTPIGDGPVLLPDNHYLSTQSALSKDPNEKRDHMVLLEFVTA AGITLGMDELYKGGGGSGVTFQSGFRKMAFPSGKVEGCMVQVTCGT TTLNGLWLDDVVYCPRHVICTSEDMLNPNYEDLLIRKSNHNFLVQAG NVQLRVIGHSMQNCVLKLKVDTANPKTPKYKFVRIQPGQTFSVLACY NGSPSGVYQCAMRPNFTIKGSFLNGSCGSVGFNIDYDCVSFCYMHHM ELPTGVHAGTDLEGNFYGPFVDRQTAQAAGTDTTITVNVLAWLYAAVI NGDRWFLNRFTTTLNDFNLVAMKYNYEPLTQDHVDILGPLSAQTGIAV LDMCASLKELLQNGMNGRTILGSALLEDEFTPFDVVRQCSGVTFQAG NATGGGGSMTSKVYDPEQRKRMITGPQWWARCKQMNVLDSFINYYD SEKHAENAVIFLHGNATSSYLWRHVVPHIEPVARCIIPDLIGMGKSGKS GNGSYRLLDHYKYLTAWFELLNLPKKIIFVGHDWGAALAFHYAYEHQ DRIKAIVHMESVVDVIESWDEWPDIEEDIALIKSEEGEKMVLENNFFVE TVLPSKIMRKLEPEEFAAYLEPFKEKGEVRRPTLSWPREIPLVKGGKPD VVQIVRNYNAYLRASDDLPKLFIESDPGFFSNAIVEGAKKFPNTEFVKV KGLHFLQEDAPDEMGKYIKSFVERVLKNEQLEGSYPYDVPDYA*
pBRET-10	MDYKDDDDKMVSKGEELFTGVVPILVELDGDVNGHKFSVSGEGEGD

	ATYGKLTLKFICTTGKLPVPWPTLVTTLSYGVQCFSRYPDHMKQHDFF KSAMPEGYVQERTIFFKDDGNYKTRAEVKFEGDTLVNRIELKGIDFKE DGNILGHKLEYNYNSHNVYIMADKQKNGIKVNFKIRHNIEDGSVQLA DHYQQNTPIGDGPVLLPDNHYLSTQSALSKDPNEKRDHMVLLEFVTA AGITLGMDELYKGGGGSVATVQSGFRKMAFPSGKVEGCMVQVTCGTT TLNGLWLDDVVYCPRHVICTSEDMLNPNYEDLLIRKSNHNFLVQAGN VQLRVIGHSMQNCVLKLKVDTANPKTPKYKFVRIQPGQTFSVLACYN GSPSGVYQCAMRPNFTIKGSFLNGSCGSVGFNIDYDCVSFCYMHHME LPTGVHAGTDLEGNFYGPFVDRQTAQAAGTDTTITVNVLAWLYAAVIN GDRWFLNRFTTTLNDFNLVAMKYNYEPLTQDHVDILGPLSAQTGIAVL DMCASLKELLQNGMNGRTILGSALLEDEFTPFDVVRQCSGVTFQNNE LSGGGGSMTSKVYDPEQRKRMITGPQWWARCKQMNVLDSFINYYDS EKHAENAVIFLHGNATSSYLWRHVVPHIEPVARCIIPDLIGMGKSGKSG NGSYRLLDHYKYLTAWFELLNLPKKIIFVGHDWGAALAFHYAYEHQD RIKAIVHMESVVDVIESWDEWPDIEEDIALIKSEEGEKMVLENNFFVET VLPSKIMRKLEPEEFAAYLEPFKEKGEVRRPTLSWPREIPLVKGGKPDV VQIVRNYNAYLRASDDLPKLFIESDPGFFSNAIVEGAKKFPNTEFVKVK GLHFLQEDAPDEMGKYIKSFVERVLKNEQLEGSYPYDVPDYA*

[1]Protein sequences of different components in the biosensors were identified as follows: green, GFP2; orange, SARS-CoV-2 3CLpro; blue, RLuc8; underscored, 3CLpro cleavage sequences. DYKDDDDK and YPYDVPDYA are the sequences of FLAG tag and HA tag, respectively.

Table S3. Experimental data of Figure 1C.

**BRET ratio**						
	pBRET-1					pBRETmut-1
GC376 concentration (μM)	0	0.32	1.6	8	40	0
Measurement 1	0.0117	0.0140	0.0195	0.0484	0.1116	0.1482
Measurement 2	0.0128	0.0178	0.0206	0.0477	0.1079	0.1652
Measurement 3	0.0119	0.0155	0.0207	0.0516	0.1112	0.1451

**410 nm channal signal**							
	pBRET-1					pBRETmut-1	Blank
GC376 concentration (μM)	0	0.32	1.6	8	40	0	0
Measurement 1	237131	208329	299462	615180	681867	569062	1043
Measurement 2	203902	275909	326871	705866	1079866	697925	1516
Measurement 3	271317	262127	336338	779238	1187572	566935	1657

**515 nm channal signal**							
	pBRET-1					pBRETmut-1	Blank
GC376 concentration (μM)	0	0.32	1.6	8	40	0	0
Measurement 1	3083	3229	6153	30046	76285	84511	320
Measurement 2	2932	5221	7056	33941	116666	115372	340
Measurement 3	3491	4305	7214	40436	132197	82300	279

Table S4. Experimental data of Figures 2A, 2B and 2C.

BRET ratio

		pBRET-1			pBRET-2			pBRET-3			pBRET-4			pBRET-5		
		Measurement 1	Measurement 2	Measurement 3	Measurement 1	Measurement 2	Measurement 3	Measurement 1	Measurement 2	Measurement 3	Measurement 1	Measurement 2	Measurement 3	Measurement 1	Measurement 2	Measurement 3
GC376 concentration (μM)	**0**	0.0130	0.0125	0.0136	0.0189	0.0220	0.0152	0.0107	0.0100	0.0100	0.0106	0.0099	0.0099	0.0209	0.0192	0.0182
	**0.15625**	0.0129	0.0125	0.0129	0.0244	0.0224	0.0182	0.0107	0.0107	0.0104	0.0103	0.0104	0.0098	0.0234	0.0247	0.0261
	**0.625**	0.0138	0.0132	0.0128	0.0315	0.0286	0.0220	0.0111	0.0105	0.0103	0.0114	0.0109	0.0108	0.0379	0.0398	0.0368
	**2.5**	0.0139	0.0127	0.0135	0.0501	0.0507	0.0392	0.0112	0.0106	0.0113	0.0116	0.0114	0.0117	0.0567	0.0644	0.0641
	**10**	0.0387	0.0532	0.0560	0.1542	0.1466	0.1388	0.0433	0.0414	0.0360	0.0601	0.0566	0.0570	0.1454	0.1460	0.1471
	**40**	0.1702	0.1570	0.1556	0.2037	0.2129	0.2011	0.1448	0.1438	0.1271	0.1481	0.1610	0.1520	0.1544	0.1574	0.1605

410 nm channal signal

		pBRET-1			pBRET-2			pBRET-3			pBRET-4			pBRET-5		
		Measurement 1	Measurement 2	Measurement 3	Measurement 1	Measurement 2	Measurement 3	Measurement 1	Measurement 2	Measurement 3	Measurement 1	Measurement 2	Measurement 3	Measurement 1	Measurement 2	Measurement 3
GC376 concentration (μM)	**0**	1234022	1151528	899887	590180	464537	477288	879563	1004427	990752	764757	775289	681650	511999	592657	640073
	**0.15625**	1298126	1342851	1093576	626435	537016	601133	1023581	1154799	1161563	836131	859874	844227	519644	649662	624884
	**0.625**	1727242	1940429	1413385	593576	576754	512711	1304554	1379853	1446160	1097906	978205	1007349	462029	568583	520016
	**2.5**	2470380	2551939	2100949	467467	412662	405402	1817391	2036083	1841547	1359178	1292842	1203698	475930	577948	479805
	**10**	1566941	1456351	1139521	263847	265237	203611	1113166	1196867	1321297	736322	818050	608763	280839	283462	250224
	**40**	317088	369666	365018	255732	174543	160397	267070	359895	309095	274990	210194	229131	134270	213273	198694
	**Blank**	2361	2641	2293	1901	1513	1090	2194	2654	2857	2639	2370	1634	1324	1804	1706

515 nm channal signal

		pBRET-1			pBRET-2			pBRET-3			pBRET-4			pBRET-5		
		Measurement 1	Measurement 2	Measurement 3	Measurement 1	Measurement 2	Measurement 3	Measurement 1	Measurement 2	Measurement 3	Measurement 1	Measurement 2	Measurement 3	Measurement 1	Measurement 2	Measurement 3
GC376 concentration (μM)	**0**	16226	14691	12405	11336	10400	7360	9653	10189	10213	8242	7842	6852	10860	11530	11810
	**0.15625**	16957	17089	14257	15470	12237	11015	11230	12489	12368	8748	9132	8368	12306	16203	16433
	**0.625**	24116	25884	18355	18843	16672	11344	14709	14657	15169	12629	10809	10967	17610	22751	19245
	**2.5**	34541	32633	28591	23517	21090	15965	20670	21714	21119	15834	14888	14188	27067	37287	30810
	**10**	60784	77607	63922	40600	38904	28212	48335	49656	47854	44235	46326	34735	40805	41316	36725
	**40**	53804	57942	56660	51901	37069	32137	38597	51562	39244	40482	33660	34710	20693	33484	31791
	**Blank**	248	307	234	205	229	102	254	206	333	159	196	131	166	201	169

BRET ratio

		pBRET-6			pBRET-7			pBRET-8			pBRET-9			pBRET-10		
		Measurement 1	Measurement 2	Measurement 3	Measurement 1	Measurement 2	Measurement 3	Measurement 1	Measurement 2	Measurement 3	Measurement 1	Measurement 2	Measurement 3	Measurement 1	Measurement 2	Measurement 3
GC376 concentration (μM)	**0**	0.0152	0.0156	0.0149	0.0220	0.0227	0.0183	0.0153	0.0135	0.0139	0.0169	0.0156	0.0173	0.0203	0.0179	0.0167
	**0.15625**	0.0225	0.0171	0.0193	0.0281	0.0282	0.0239	0.0166	0.0155	0.0148	0.0170	0.0187	0.0178	0.0233	0.0234	0.0219
	**0.625**	0.0276	0.0249	0.0268	0.0434	0.0367	0.0340	0.0219	0.0172	0.0177	0.0256	0.0247	0.0263	0.0368	0.0316	0.0309
	**2.5**	0.0568	0.0434	0.0481	0.0738	0.0616	0.0521	0.0374	0.0356	0.0332	0.0412	0.0436	0.0439	0.0662	0.0679	0.0582
	**10**	0.1430	0.1380	0.1457	0.1231	0.1326	0.1274	0.1272	0.1263	0.1264	0.1302	0.1315	0.1245	0.1297	0.1231	0.1289
	**40**	0.1549	0.1637	0.1461	0.1298	0.1461	0.1375	0.1396	0.1382	0.1316	0.1472	0.1501	0.1523	0.1237	0.1160	0.1241

410 nm channal signal

		pBRET-6			pBRET-7			pBRET-8			pBRET-9			pBRET-10		
		Measurement 1	Measurement 2	Measurement 3	Measurement 1	Measurement 2	Measurement 3	Measurement 1	Measurement 2	Measurement 3	Measurement 1	Measurement 2	Measurement 3	Measurement 1	Measurement 2	Measurement 3
GC376 concentration (μM)	**0**	547248	482208	445856	233952	323964	444434	627970	588543	630641	489600	595209	576136	437500	432661	395263
	**0.15625**	535509	475262	454595	376256	320837	351212	660007	646006	586368	562965	663910	583957	392545	372361	352697
	**0.625**	558333	508110	471909	229665	269666	325034	683542	683651	624476	477967	583392	482931	343591	338509	323550
	**2.5**	467092	511607	443973	241141	184994	262223	654140	530182	551924	398200	430743	358479	287913	226924	180487
	**10**	258684	225573	223437	171368	134529	132983	268175	230820	257923	240314	288683	212303	208061	184788	173127
	**40**	218365	132623	172381	112006	121109	128929	241809	195123	227171	224353	249342	158081	181962	148924	159990
	**Blank**	1601	1262	991	1470	1558	1821	1885	1484	1554	1595	1677	1478	1535	1182	958

515 nm channal signal

		pBRET-6			pBRET-7			pBRET-8			pBRET-9			pBRET-10		
		Measurement 1	Measurement 2	Measurement 3	Measurement 1	Measurement 2	Measurement 3	Measurement 1	Measurement 2	Measurement 3	Measurement 1	Measurement 2	Measurement 3	Measurement 1	Measurement 2	Measurement 3
GC376 concentration (μM)	**0**	8553	7668	6722	5295	7588	8274	9722	8191	8849	8390	9554	10138	9052	7863	6674
	**0.15625**	12264	8250	8882	10728	9268	8536	11074	10231	8802	9656	12665	10550	9279	8831	7781
	**0.625**	15604	12799	12742	10086	10109	11151	15030	11990	11144	12298	14677	12863	12756	10791	10038
	**2.5**	26684	22326	21435	17866	11564	13731	24531	19061	18389	16469	19023	15837	19152	15464	10535
	**10**	37012	31115	32533	21088	17900	16888	34002	29214	32540	31211	38043	26433	26974	22731	22272
	**40**	33827	21672	25148	14526	17733	17657	33624	27011	29808	32901	37482	24033	22510	17277	19821
	**Blank**	261	162	113	181	272	174	124	241	126	122	303	178	184	135	83

Table S5. Experimental data of Figure 2D.

BRET ratio

		0.8 μg DNA/million cells					1.2 μg DNA/million cells					1.6 μg DNA/million cells					2.0 μg DNA/million cells				
		Measurement 1	Measurement 2	Measurement 3	Measurement 4	Measurement 5	Measurement 1	Measurement 2	Measurement 3	Measurement 4	Measurement 5	Measurement 1	Measurement 2	Measurement 3	Measurement 4	Measurement 5	Measurement 1	Measurement 2	Measurement 3	Measurement 4	Measurement 5
GC376 concentration (μM)	**40**	0.1103	0.1213	0.1045	0.1248	0.1301	0.1117	0.1263	0.1048	0.1119	0.1272	0.1138	0.1236	0.1037	0.1150	0.1245	0.1033	0.1255	0.0986	0.1084	0.1225
	**20**	0.1102	0.1222	0.1009	0.1275	0.1213	0.1095	0.1244	0.0984	0.0816	0.1300	0.1105	0.1265	0.0951	0.0674	0.1295	0.1106	0.1279	0.1052	0.0925	0.1256
	**10**	0.1160	0.1233	0.1054	0.1317	0.1300	0.1185	0.1147	0.0967	0.0765	0.1242	0.1103	0.1274	0.0921	0.0655	0.1220	0.1130	0.1177	0.1037	0.0874	0.1148
	**5**	0.1119	0.1065	0.1033	0.1230	0.1272	0.0972	0.0960	0.0936	0.0869	0.1096	0.0936	0.0872	0.0841	0.0784	0.1129	0.0906	0.0743	0.0873	0.1076	0.0930
	**2.5**	0.0890	0.0810	0.0805	0.0724	0.1061	0.0680	0.0521	0.0562	0.0414	0.0862	0.0433	0.0469	0.0549	0.0393	0.0782	0.0370	0.0388	0.0510	0.0454	0.0662
	**1.25**	0.0610	0.0588	0.0522	0.0426	0.0621	0.0390	0.0418	0.0358	0.0238	0.0434	0.0278	0.0292	0.0356	0.0303	0.0387	0.0179	0.0241	0.0303	0.0219	0.0300
	**0.625**	0.0409	0.0435	0.0255	0.0271	0.0356	0.0243	0.0301	0.0204	0.0184	0.0275	0.0160	0.0217	0.0200	0.0183	0.0268	0.0127	0.0176	0.0186	0.0168	0.0191
	**0.3125**	0.0306	0.0346	0.0216	0.0216	0.0290	0.0185	0.0215	0.0194	0.0173	0.0255	0.0140	0.0205	0.0174	0.0185	0.0220	0.0116	0.0158	0.0155	0.0144	0.0189
	**0.15625**	0.0257	0.0297	0.0178	0.0194	0.0210	0.0159	0.0218	0.0145	0.0141	0.0190	0.0122	0.0181	0.0141	0.0150	0.0179	0.0110	0.0148	0.0137	0.0127	0.0145
	**0**	0.0171	0.0204	0.0118	0.0153	0.0141	0.0124	0.0164	0.0118	0.0129	0.0143	0.0106	0.0156	0.0113	0.0127	0.0144	0.0101	0.0119	0.0109	0.0113	0.0133

410 nm channal signal

		0.8 μg DNA/million cells					1.2 μg DNA/million cells					1.6 μg DNA/million cells					2.0 μg DNA/million cells				
		Measurement 1	Measurement 2	Measurement 3	Measurement 4	Measurement 5	Measurement 1	Measurement 2	Measurement 3	Measurement 4	Measurement 5	Measurement 1	Measurement 2	Measurement 3	Measurement 4	Measurement 5	Measurement 1	Measurement 2	Measurement 3	Measurement 4	Measurement 5
GC376 concentration (μM)	**40**	176013	199748.5	374183.5	418491	211782	200463	137447.5	423534.5	455419	525676	159289	264964.5	464404.5	601097	560332	285091.5	300523	468787.5	345145	621388
	**20**	180364	215900	352589.5	446831	356585	254546	228069	423223.5	423245	497238	201421.5	333135	374353	474057	606657	285274.5	381061.5	431289.5	392580	848114
	**10**	113620.5	168815.5	211881.5	469532	397226	159516.5	228793.5	231636	275208	350947	148389	278456	306580.5	292286	658560	276629.5	469351	290549	287525	795563
	**5**	115296.5	206619	223720	680133	414585	189951	290387	244063.5	569415	521453	225175.5	493747.5	340102	292914	770524	408221.5	826516	413621.5	487545	1287088
	**2.5**	130848.5	275292.5	259275.5	1090848	462567	205195.5	379067	299189.5	1457687	635611	320586.5	642703.5	416796.5	726177	912197	619301	1051115	527399	868446	1155199
	**1.25**	195673.5	248731	295157.5	1206607	650832	325679.5	390822.5	348128.5	504187	928067	353803	766150	478523	539211	1269814	1071483	1226237	713764.5	1176284	1577702
	**0.625**	269460	333241	434321.5	1304494	955027	514296	476456.5	574078.5	820073	1249502	793634	926150	716593.5	1284701	1494075	1496073	1332125	1029798	1583770	1978855
	**0.3125**	278345	337108	535567.5	1669325	871227	528700	658718	656579	1096121	1481419	825278.5	925973.5	882520.5	978737	1534966	1472552	1385189	1223740	1686743	2174349
	**0.15625**	322640	372615.5	630055.5	1459479	1001347	593337	592214	835440	1168593	1774757	856749.5	929602	879900	995747	1708868	1517177	1282589	1133399	1674622	2030165
	**0**	428903.5	483341	832829.5	1310795	1064073	686641.5	617382.5	921304.5	1320987	1691079	950609	808978.5	935851.5	1654732	1827282	1520391	1261107	1165243	1920702	2136159
	**Blank**	971	1061.5	1541	1352	1592	1505.5	1528	2020	2054	2149	1770	1840.5	2266.5	2464	2646	1589.5	1845	2175.5	2499	2738

515 nm channal signal

		0.8 μg DNA/million cells					1.2 μg DNA/million cells					1.6 μg DNA/million cells					2.0 μg DNA/million cells				
		Measurement 1	Measurement 2	Measurement 3	Measurement 4	Measurement 5	Measurement 1	Measurement 2	Measurement 3	Measurement 4	Measurement 5	Measurement 1	Measurement 2	Measurement 3	Measurement 4	Measurement 5	Measurement 1	Measurement 2	Measurement 3	Measurement 4	Measurement 5
GC376 concentration (μM)	**40**	19420.5	24226.5	39009.5	52172	27405	22350.5	17337.5	44387.5	50797	66742	18042	32675.5	48091.5	68966	69591	29384.5	37614	46152	37321	75947
	**20**	19878	26391.5	35507	56894	43137	27836	28356.5	41647.5	34427	64544	22181.5	42042.5	35591	31910	78416	31456.5	48621.5	45255	36257	106324
	**10**	13175.5	20820.5	22260	61741	51498	18848.5	26240	22407.5	20953	43481	16285	35394.5	28205.5	19107	80170	31163	55128	30041	25096	91223
	**5**	12908	22017.5	23042	83585	52607	18443	27906.5	22839.5	49380	57065	21013	43050	28605.5	22903	86846	36915.5	61403	36053.5	52349	119592
	**2.5**	11677	22330.5	20821.5	78996	48993	13977	19830.5	16884	60285	54767	13908	30202.5	22954.5	28552	71280	22974.5	40856	26904	39469	76466
	**1.25**	11984	14696	15401.5	51414	40377	12759.5	16423	12586	11993	40326	9900.5	22459	17127.5	16410	49241	19243	29674	21682.5	25894	47424
	**0.625**	11097	14579	11101	35351	34031	12586.5	14482	11867.5	15122	34438	12767	20187.5	14464	23607	40203	19088	23585	19274	26704	37908
	**0.3125**	8598	11744	11640.5	36080	25258	9857.5	14265	12891.5	19000	37825	11674	19105.5	15514.5	18159	33932	17176	21970	19012.5	24384	41178
	**0.15625**	8391.5	11147.5	11272.5	28439	21097	9559.5	13052.5	12237.5	16559	33766	10579	16906.5	12545	15003	30639	16785	19100	15569	21397	29550
	**0**	7437.5	9961.5	9906	20181	15054	8604.5	10290.5	10999	17091	24337	10217	12705	10779.5	21132	26383	15479.5	15166.5	12840	21930	28648
	**Blank**	113.5	130.5	84.5	98	65	122.5	166.5	194.5	65	168	112	146.5	189.5	121	179	93.5	124	124	177	169

Table S6. Experimental data of Table 1.

BRET ratio

		GC376			Boceprevir			11a			13b		
		Measurement 1	Measurement 2	Measurement 3	Measurement 1	Measurement 2	Measurement 3	Measurement 1	Measurement 2	Measurement 3	Measurement 1	Measurement 2	Measurement 3
**Inhibitor concentration (μM)**	0	0.0108	0.0108	0.0110	0.0117	0.0124	0.0111	0.0117	0.0126	0.0132	0.0145	0.0126	0.0131
	0.12	0.0118	0.0117	0.0110	0.0120	0.0120	0.0119	0.0133	0.0147	0.0149	0.0156	0.0142	0.0129
	0.4	0.0128	0.0129	0.0147	0.0127	0.0122	0.0111	0.0153	0.0170	0.0173	0.0151	0.0138	0.0115
	1.2	0.0178	0.0182	0.0171	0.0137	0.0118	0.0125	0.0273	0.0302	0.0303	0.0151	0.0139	0.0125
	4	0.0886	0.0795	0.0769	0.0128	0.0131	0.0134	0.0599	0.0493	0.0478	0.0160	0.0146	0.0133
	12	0.1266	0.1308	0.1240	0.0139	0.0148	0.0152	0.1188	0.1254	0.1302	0.0189	0.0186	0.0167
	40	0.1212	0.1220	0.1293	0.0801	0.0823	0.0855	0.1387	0.1282	0.1311	0.0706	0.0754	0.0735
	120	0.1224	0.1166	0.1151	0.1350	0.1350	0.1245	0.1157	0.1230	0.1247	0.1187	0.1195	0.1131

410 nm channal signal

		GC376			Boceprevir			11a			13b		
		Measurement 1	Measurement 2	Measurement 3	Measurement 1	Measurement 2	Measurement 3	Measurement 1	Measurement 2	Measurement 3	Measurement 1	Measurement 2	Measurement 3
**Inhibitor concentration (μM)**	0	543414	760385	775980	579480	716719	682130	630687	1067466	954683	821340	754520	766753
	0.12	578191	719633	737095	595713	690360	652259	585997	964515	839140	666072	793269	736956
	0.4	540450	719705	654227	541547	584623	648167	569398	926440	905528	676838	711126	728500
	1.2	409477	498414	566838	424746	529770	560261	363138	551192	610633	802087	713698	753889
	4	147808	203199	219620	603440	620984	607605	221963	407828	419719	805094	719217	623176
	12	63714	95777	100730	566851	598685	659855	94899	202511	155832	933637	834692	728761
	40	59834	77515	85257	315421	293353	311020	42621	107057	84365	378470	250933	216433
	120	53997	68132	68084	78713	98451	99369	13338	29103	18042	61734	53595	33251
	Blank	1204	1994	2124	2256	2191	1787	1849	2549	2737	3256	3035	2495

515 nm channal signal

		GC376			Boceprevir			11a			13b		
		Measurement 1	Measurement 2	Measurement 3	Measurement 1	Measurement 2	Measurement 3	Measurement 1	Measurement 2	Measurement 3	Measurement 1	Measurement 2	Measurement 3
**Inhibitor concentration (μM)**	0	5895	8282	8600	6875	8965	7657	7463	13580	12734	12170	9675	10223
	0.12	6889	8523	8229	7224	8336	7796	7879	14314	12613	10625	11407	9708
	0.4	6971	9337	9731	6953	7177	7264	8770	15886	15825	10441	10018	8579
	1.2	7325	9139	9793	5912	6299	7059	9944	16721	18582	12318	10090	9588
	4	13049	16097	16843	7832	8206	8220	13266	20117	20108	13104	10695	8447
	12	7980	12373	12338	7977	8874	10058	11135	25205	20116	17886	15724	12313
	40	7171	9320	10861	25180	24031	26532	5741	13534	10891	26743	18915	15934
	120	6528	7820	7705	10432	13072	12238	1414	3400	2097	7208	6259	3691
	Blank	66	110	114	109	73	87	85	134	189	269	215	213

Table S7. Experimental data of Figure S1.

**BRET ratio**				
	Measurement 1	Measurement 2	Measurement 3	Measurement 4
0.8 μg DNA/million cells	0.1212	0.1155	0.1163	0.1273
1.2 μg DNA/million cells	0.1172	0.1143	0.1257	0.1186
1.6 μg DNA/million cells	0.1436	0.1230	0.1391	0.1275
2.0 μg DNA/million cells	0.1344	0.1410	0.1362	0.1203

**410 nm channel signal**						Blank			
	Measurement 1	Measurement 2	Measurement 3	Measurement 4		Measurement 1	Measurement 2	Measurement 3	Measurement 4
0.8 μg DNA/million cells	43101	65306	36231	47579		708	1039	784	994
1.2 μg DNA/million cells	113257	95433	82872	105243		1213	1746	1189	1386
1.6 μg DNA/million cells	145827	129117	144251	129782		1888	1973	1687	2516
2.0 μg DNA/million cells	160949	119567	157937	125287		1839	1562	2464	2001

**515 nm channel signal**						Blank			
	Measurement 1	Measurement 2	Measurement 3	Measurement 4		Measurement 1	Measurement 2	Measurement 3	Measurement 4
0.8 μg DNA/million cells	5242	7548	4200	5978		102	128	79	50
1.2 μg DNA/million cells	13229	10905	10362	12390		101	192	93	72
1.6 μg DNA/million cells	20816	15780	19973	16435		151	143	136	213
2.0 μg DNA/million cells	21522	16707	21327	14895		131	67	149	66
